# Justicidin B: A Promising Bioactive Lignan †

**DOI:** 10.3390/molecules21070820

**Published:** 2016-06-23

**Authors:** Shiva Hemmati, Hassan Seradj

**Affiliations:** 1 Department of Pharmaceutical Biotechnology, School of Pharmacy, Shiraz University of Medical Sciences, P. O. Box 71345-1583 Shiraz, Iran; 2 Pharmaceutical Sciences Research Center, Shiraz University of Medical Sciences, P. O. Box 71345-3119 Shiraz, Iran; 3 Department of Pharmacognosy, School of Pharmacy, Shiraz University of Medical Sciences, P. O. Box 71345-1583 Shiraz, Iran; serajh@sums.ac.ir

**Keywords:** lignan, arylnaphthalene, justicidin B, distribution, chemistry, biosynthesis, biotechnological production, biological activity, synthesis, chromatography

## Abstract

Adverse effects and drug resistance to the current onchopharmacologicals have increased the demand for alternative novel therapeutics. We herein introduce justicidin B, an arylnaphthalen lignan isolated from different plant origins, especially *Justicia*, *Phyllanthus*, *Haplophyllum* and *Linum* species. This cyclolignan exhibits a wide array of biological properties ranges from piscicidal to antifungal, antiviral and antibacterial activities. Activity against *Trypanosoma brucei* makes justicidin B a potential antiprotozoal agent for the treatment of neglected tropical diseases. Pharmacological properties like antiplatelet, anti-inflammatory and bone resorption inhibition have been also attributed to justicidin B. This compound is a potent cytotoxic substance on several cell lines, especially chronic myeloid and chronic lymphoid leukemia. Pharmacological values, natural variation, as well as biotechnological production of justicidin B by plant cell, tissue and organ culture are also described in this review. Chemical characteristics and chromatographic methods to identify justicidin B and its biosynthetic pathway have been discussed. Different approaches to the total synthesis of justicidin B are compared. This review would shed light on the role of justicidin B as an intriguing natural compound and provides a chance to optimize conditions for industrial applications.

## 1. Introduction

**Scheme 1:**
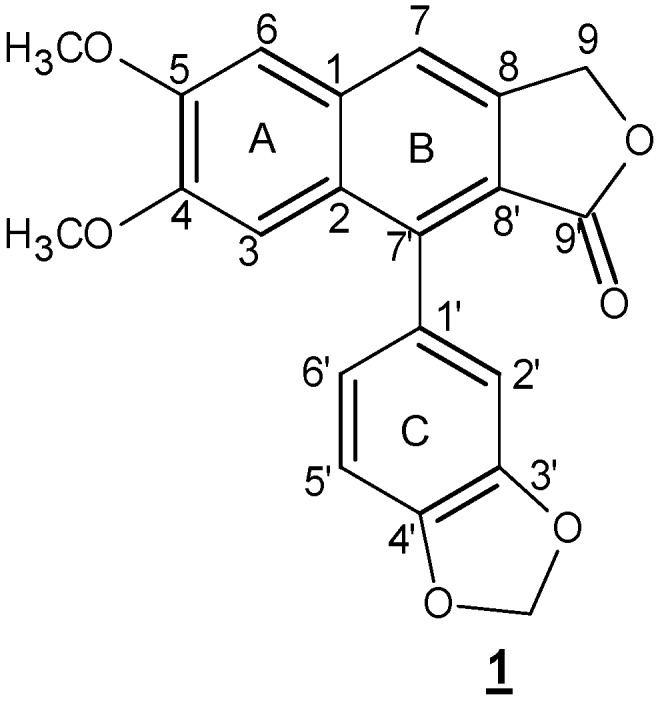
Chemical structure of justicidin B. Two phenylpropanoid units are linked via 8-8′ carbon atoms.

Lignans are phenylpropanoid (C6C3) inter-unit linkages, which are mainly classified into eight different subgroups [1,2]. These widespread, specialized metabolites are, not only important as plant defense machinery, but also valuable for human health [3]. Currently, the most applicable lignan is podophyllotoxin (the aryltetralin compound), which has been used as a precursor for the semi-synthesis of etoposide in the pharmaceutical industry [4]. Overgrowing resistance to current therapeutics has urged scientists to screen novel pharmaceuticals. Justicidin B (**1**) (Scheme 1), classified as an arylnaphthalene lignan, has intriguing pharmacological properties [5]. This planar biaryl molecule has attracted interest to be considered as a potential lead compound for novel therapeutics.

In this review, the distribution, biosynthesis, biotechnological production, chemical, biological and pharmacological properties of justicidin B are described. Analytical methods for the isolation and quantification of the molecule are discussed and various strategies for the chemical synthesis of justicidin B are compared. Peer reviewed articles on justicidin B were retrieved from Google Scholar, Pubmed, Scopus and ISI Web of Knowledge until from 1960 to April 2016.

## 2. Chemistry

4,5-dimethoxy-3′,4′-methylenedioxy-2,7′-cycloligna-7,7′-dieno-9,9′-lactone (justicidin B) C_21_H_16_O_6_: colorless solid; mp 234–237 °C [6] (235–238 °C [7]); UV (PDA detector of HPLC) λ_max_ at 258, 296, 310 nm; ^1^H-NMR (CDCl_3_) δ 3.81 (s, 3H), 4.05 (s, 3H), 5.37 (AB, 2H, Δδ = 0.93, *J* ≈ 14 Hz), 6.07 (AB, 2H, Δδ = 12.48, *J* = 1.46 Hz), 6.79 (dd, 1H, *J* = 7.80, 1.48 Hz), 6.85 (s, 1H), 6.96 (d, 1H, *J* = 7.82 Hz), 7.10 (s, 1H), 7.18 (s, 1H), 7.69 (s, 1H). GC-EI-MS *m*/*z* 364 [M]^+^ (100), 335 (4), 319 (6), 305 (11), 291 (8), 277 (19), 167 (18), 163 (17); IR (KBr) 1760, 1620, 931, 873, 811 cm^−1^ [6,7,8]. Detailed assignment of the NMR data is presented in Table 1.

### Solubility

It has been shown that justicidin B is poorly soluble in water but completely soluble in pure DMSO up to 1.5 mg/mL. Its solubility in water or culture medium is 0.05 mg/mL [10]. Wang et al. were able to dissolve 0.42 mg/mL justicidin B in HPLC-grade acetonitrile [11].

## 3. Distribution

In 1965, for the first time, justicidin B—a fish killing agent among native Taiwanese—was identified as the active principle of *Justicia hayatai* var. *decumbens* (Acanthaceae), in which the methylenedioxy bridge was mistakenly interpreted by the authors [12]. Later, in 1967, Govindachari et al. revised the structure of justicidins isolated from *Cleistanthus collinus* (Euphorbiacea) [13]. The concise chemical structure of justicidin B was also described by Okigawa et al. [7]. Accumulation of justicidin B has been reported in *Justicia* (Acanthaceae), *Phyllanthus* (Euphorbiaceae), *Haplophyllum* (Rutaceae) and *Linum* (Linaceae) species.

*Justicia* with 600 species is a rich lode of justicidin B [14]. In addition to *J. hayatai*, analysis of *Justicia procumbens* [7,15], *Justicia pectoralis* [16], *Justicia ciliate* [17], and *Justicia purpurea* [18] whole plants have shown the accumulation of justicidin B.

*Haplophyllum*s, with about 70 species, are another origin to mine justicidin B [19]. This compound has been detected in *Haplophyllum obtusifolium* [20,21], *Haplophyllum dauricum* (epigeal parts) [22], *Haplophyllum buxbaumii* (aerial parts) [23,24], *Haplophyllum tuberculatum* (flowering aerial parts) [25,26], *Haplophyllum cappadocicum* (whole plant) [27] and *Haplophyllum patavinum* [28].

*Phyllanthus*, with more than 700 species, is another major source of justicidin B [29]. *Phyllanthus acuminatus* (root) [30,31], *Phyllanthus anisolobus* (leaves and twigs: 0.6 DW%) [32], *Phyllanthus myrtifolius* (aerial part) [33,34], *Phyllanthus piscatorum* (aerial part: 3–4 DW%) [10] and *Phyllanthus polyphyllus* (whole plant) [35] all accumulate justicidin B.

Secondary metabolites may act as a chemotaxonomic marker. The presence of dominant arylnaphthalene lignans like justicidin B in the sections Linum and Dasyllinum of the Linaceae family has shown a good agreement with molecular and morphological systematics [36]. Flowering aerial parts of the section Linum including *Linum austriacum*, *Linum lewissi*, *Linum perenne*, *L. perenne* ssp. *Diamant*, *L. perenne* ssp. *Himelszelt*, *Linum alpinum*, *Linum leonii*, *Linum meletonis*, *Linum altaicum* and *Linum bungei* accumulate justicidin B. *Linum hirsutum* and *Linum stelleroides* from the section Dasillinum showed comparable amount of justicidin B to the section Linum. However, trace amounts of justicidin B could be detected in *Linum linearifolium* (section syllinum) and *Linum trigynum* (section Linopsis) [36,37,38]. The presence of justicidin B in the seeds of *Linum* species has been investigated by Schmidt et al. [39]. Seeds of *Linum pallescens* and *L. perenne* ssp. *extraaxillare* from the section Linum contain justicidin B as much as 1–3 mg/g seed weight while seeds of *L. altaicum*, *Linum komarowii*, *L. leoni*, *L. lewissi*, *L. perenne* ssp. *perenne*, *L. perenne* ssp. *anglicum* and *L. perenne* ssp. *alpinum* accumulate 0.1–1 mg/g seed weight justicidin B. *Linum decumbens* seeds contain less than 0.1 mg/g seed weight justicidin B. *L. stelleroides* from the section Dasillinum has 0.1–1 mg/g seed weight justicidin B. Justicidin B content in the seeds has shown a good correlation with the aerial parts of the same plant except for *L. hirsutum*. Seeds of *Sesbania drummondii* contain 9 × 10^−7^ DW% justicidin B [40]. The presence of justicidin B has been reported from the marine microorganism *Nocardia* sp. ALAA 2000 [41]. Effect of geographical origin on the accumulation of justicidin B in single plant species is also studied [11]. Twenty samples of *J. procumbens* have been analyzed. All samples contained varied concentration of justicidin B from 0.010 to 0.269 mg/g DW.

## 4. Total Synthesis of Justicidin B

**Scheme 2:**
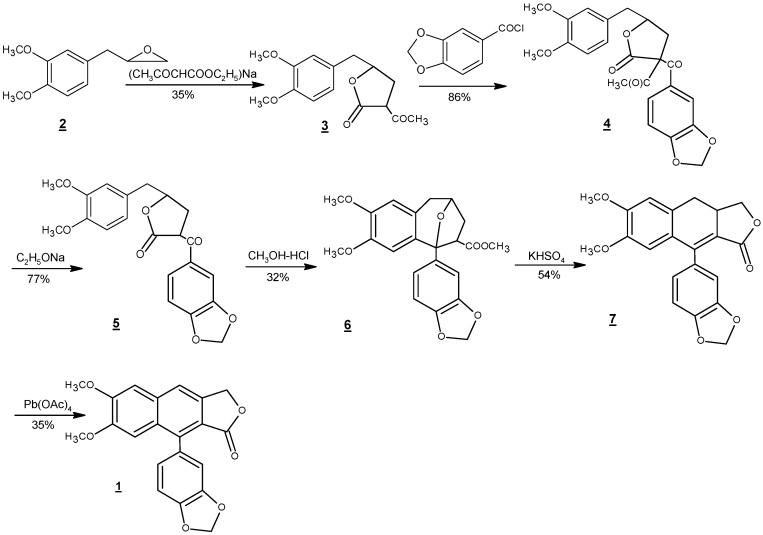
Total synthesis of justicidin B (**1**) from methyleugenoloxide (**2**) with an overall yield of 1%–2% [42].

There have been numerous reports towards the synthesis of justicidin B, other members of its family, and their non-natural derivatives [42,43,44,45,46,47,48,49,50,51,52,53,54,55,56,57,58,59]. The first total synthesis of justicidin B was conducted by Munakata et al. in 1967 [42]. Starting from methyleugenoloxide (**2**), as illustrated in Scheme 2, this six-step route focused on the construction of the naphthalene ring of justicidin B. This synthetic strategy of devising naphthalene ring closure as the key step has been utilized in all synthetic routes to this natural product thereafter. The condensation of **2** with sodium ethyl acetoacetate and then piperonyloyl chloride resulted in the formation of **3** and **4**, respectively. Deacetylation of **4** followed by acid catalyzed ring formation to give compound **6**. To complete the construction of justicidin B framework, compound **6** was treated with potassium hydrogen sulfate at 180 °C to afford the rearranged product dihydrojusticidin B (**7**). Finally, **1** was synthesized in 35% yield via the oxidation of **7** using lead tetraacetate Pb(OAc)_4_ with an overall yield of 1%–2% from **2**.

Subsequently, there have been many successful synthetic efforts towards the total synthesis of justicidin B. Although several of these reports directed at making a series of justicidin natural and non-natural analogs along with justicidin B, they feature a variety of synthetic strategies, such as photocyclization [44], cationic cyclization [47], benzannulation [52,53], radical cyclization [55], palladium-promoted cyclization [56] and intramolecular Diels-Alder reactions [57,58] for the construction of naphthalene ring as the key step in all the reported syntheses.

**Scheme 3:**
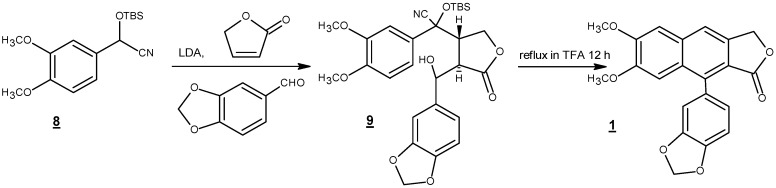
Formation of the naphthalene ring after tandem conjugate addition-aldol reaction [47].

Among these reports, two short and most efficient syntheses are presented here. In 1990, Ogiku et al. published a two-step very convergent route to justicidin B starting from readily available arylcyanohydrin (**8**) (Scheme 3) [47]. Hence, conjugate addition, followed by one-pot acid-catalyzed cationic cyclization using trifluoroacetic acid, yielded justicidin B in 58% yield over two steps. It should be mentioned that compound **9** was taken to the cyclization step without purification.

**Scheme 4:**
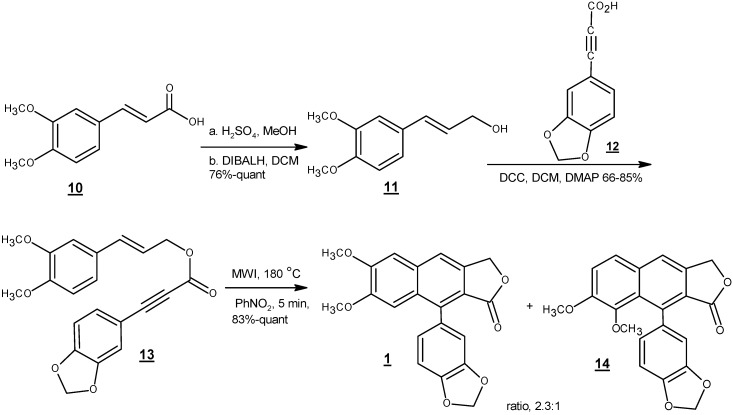
Intramolecular dehydro Diels-Alder reaction of styrenyl precursor [58].

In 2014, Kocsis et al. reported an efficient four-step route to justicidin B starting from commercially available dimethoxycinnamic acid (**10**) (Scheme 4) [58]. Reduction of carboxylic acid to alcohol (**11**) followed by esterification with compound **12** to give compound **13** set the stage for the key cyclization step, a microwave-assisted intramolecular dehydro Diels-Alder cyclization, to directly afford justicidin B and isojusticidin B (**14**) in high yield and 2.3:1 ratio of isomers favoring justicidin B.

## 5. Production of Justicidin B Using Biotechnology

Since chemical synthesis of justicidin B does not seem to be economically reasonable, biotechnological production using in vitro cell culture is an intriguing alternative for scale up purposes [60,61]. Consistent production of metabolites regardless of geographical limitations, high growth rate and virus free clones are some advantages of in vitro culture over cultivated or wild type plants [62,63]. Production of justicidin B by in vitro cultures has been reported for shoot cultures of *H. patavinum* grown in hormone free Murashige and Skoog (MS) medium (12 h photoperiod), as well as callus and suspension cultures [28,64]. Mohagheghzadeh et al. have assessed the accumulation of justicidin B in different in vitro cultures of *L. austriacum* [8]. Hairy roots (after 30 days), roots (after 30 days), suspension (after 12 days) and callus (after 30 days) derived seedlings of *L. austriacum* could produce 16.9, 12.5, 6.7 and 2.9 mg/g DW justicidin B respectively. They have evaluated the effect of medium composition on the yield of justicidin B production. A basal MS medium with 0.4 mg/lit naphthalene acetic acid (α-NAA) as the only plant growth regulator was compared with the MS medium containing 1 mg/lit α-NAA, 0.5 mg/lit kinetin, 0.5 mg/lit 2,4-dichlorophenoxy acetic acid (2,4-D) and 15% (*v*/*v*) coconut milk. Using α-NAA as the sole regulator resulted in 3.7 times higher accumulation of justicidin B in cell suspension cultures of *L. austriacum*. The accumulation of justicidin B in 9-day-old cell suspension culture of *L. austriacum* ssp. *euxinum* (0.50–0.96 DW%), *L. lewisii* (0.16–0.30 DW%) and *L. altaicum* (0.92–0.96 DW%) has been reported [65]. Screening of Bulgarian flora in vitro cultures has shown numerous species, producing justicidin B [66]. Callus (light grown) and suspension cultures (dark grown) of *L. narbonense* accumulate 1.57 and 0.09 mg/g DW justicidin B [67,68]. Callus culture of *L. leonii* contains 2.23 mg/g DW justicidin B. Cultures were seedling derived and were grown in MS media containing indole acetic acid (IAA) (0.2 mg/lit), 2,4-D (0.1 mg/lit) and kinetin (2 mg/lit). The amount of justicidin B in hairy root cultures of *L. leonii* was 10.8 mg/g DW, which is five-fold higher than the callus culture [69,70]. Callus and suspension cultures of *L. campanulatum* which were grown in similar conditions with *L. leonii* contained 0.40 and 1.41 mg/g DW justicidin B, respectively [71]. The presence of justicidin B has been reported in the shoot and callus culture of *L. glaucum* [72]. Seedling-derived calli were used to initiate dark grown cell suspension culture of *L. perenne* ssp. *Himmelszelt* on MS medium supplemented with 0.4 mg/lit α-NAA [73]. The characterization of suspension cultures during 14 days has shown that the maximum amount of justicidin B is 23 mg/g DW in 8–10 days. Hairy root cultures of *L. perenne* were initiated from in vitro shoot cultures and accumulated up to 37 mg/g DW justicidin B. This value is at least 2–3 times higher than *L. austriacum* in vitro cultures [74,75]. As a result, it is concluded that type of culture (for example, differentiated tissues and organs produced higher justicidin B) and appropriate culture condition (for example, role of phytohormones on the amount of justicidin B accumulation) are critical factors on the sustainable bioproduction of justicidin B. Since in vitro amount of justicidin B is not satisfactory yet, metabolic engineering of the biosynthetic pathway leading to this compound is the next approach for commercial production [76].

## 6. Biosynthesis

**Scheme 5:**
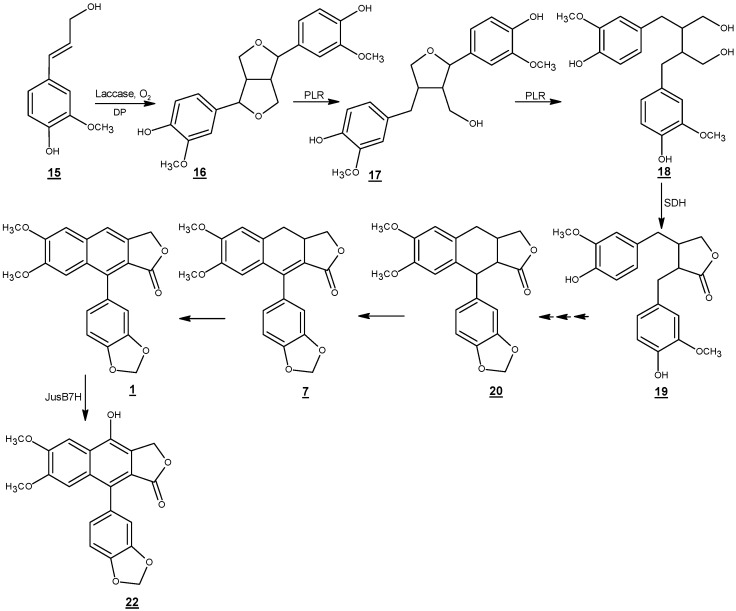
Proposed biosynthetic pathway of justicidin B. DP: dirigent protein; PLR: pinoresinol lariciresinol reductase; SDH: secoisolariciresinol dehydrogenase; Jus B 7H: justicidin B 7-hydroxylase [77].

Justicidin B biosynthesis has been studied in in vitro cultures of *L. perenne* ssp. *Himmelszelt* et al. (Scheme 5) [73,77]. The first steps in the biosynthetic pathway of justicidin B are similar to the lignan biosynthesis in several other plant species. *E*-coniferyl alcohol (**15**) derived from the monolignol pathway is converted to phenoxy radical intermediates by one electron oxidation through peroxidase/laccase dehydrogenative enzymes [78,79]. Then, the resonance stabilized phenoxy radicals might undergo different dimerization modes in vitro which results in a mixture of racemates [1]. However, the coupling of monolignol derivatives in vivo is usually regio-, diastereo-, and enantioselective [80]. The stereoselective coupling of phenoxy radicals is controlled by the involvement of a dirigent protein which does not have any catalytic activity [81]. While (+)-pinoresinol forming dirigent proteins guide free radical species to be coupled via *si*-*si* face, (−)-pinoresinol forming dirigent proteins mediate free radical couplings via *re*-*re* face [82]. This results in the formation of pinoresinol with *8R*,*8*′*R* and *8S*,*8*′*S* configurations at the streogenic centers, respectively [83].

An NADPH-dependent bifunctional enzyme named pinoresinol lariciresinol reductase (PLR-Lp1) converts pinoresinol (**16**) to secoisolariciresinol (**18**) via lariciresinol (**17**). It has been shown in several plants that there are at least two copies of the *plr* gene in the genome of the corresponding species [84,85]. One gene usually encodes a PLR enzyme which is enantiospecific for the conversion of *8S*,*8*′*S*-(−)-pinoresinol via *8S*,*8*′*S*-(−)-lariciresinol to *8S*,*8*′*S* -(+)-secoisolariciresinol the other gene is responsible for encoding an enzyme which converts *8R*,*8*′*R*-(+)-pinoresinol via *8R*,*8*′*R*-(+)-lariciresinol to *8R*,*8*′*R*-(−)-secoisolariciresinol. In vitro assays of PLR-Lp1, which involves in the biosynthetic pathway of justicidin B (without any chiral center) showed that both enantiomers of pinoresinol were converted to (+)- and (−)-lariciresinol. Similar behavior was observed in the formation of (+)- and (−)-secoisolariciresinol. However, PLR-Lp1 displayed preference for the (+)- enantiomer (RR configuration) in the first reaction step and (–)- enantiomer (SS configuration) in the second reaction step. Thus, PLR-Lp1 was introduced as the first PLR with opposite enantiospecificity toward pinoresinol and lariciresinol. Although, the involvement of PLR-Lp1 in the biosynthesis of justicidin B has been demonstrated by RNAi approach; presence of at least a second more enantiospecefic PLR encoding gene, in the genome of *L. perenne* for the biosynthesis of justicidin B has been postulated [74].

Secoisolariciresinol is converted to matairesinol (**19**) by the enzyme secoisolariciresinol dehydrogenase (SDH) [86]. Later steps including closure and aromatization of ring B, methylenedioxy bridge formation on ring C and 4 and 5-*O*-methylations have been less investigated. Formation of justicidin B has been proposed from 7(8)- 7′(8′)-tetrahydrojusticidin B (**20**) and 7(8)-dihydrojusticidin B (collinusin) (**7**) [77]. A cytochrome P450 enzyme (justicidin B 7-hydroxylase) incorporates a hydroxyl group at the C-7 position and converts justicidin B (**1**) to diphyllin (**22**) [77]. The enzyme has a *Km* value about 3.9 ± 1.3 µM. Diphyllin is then glycosylated to become more water soluble for storage in the vacuole.

## 7. Analysis

### 7.1. HPLC and HPLC-MS Analysis of Justicidin B

High Performance Liquid Chromatography (HPLC) is a robust technique to identify and isolate natural products [87]. Using the type of fragmentation and cleavage pattern by mass spectrometry to discriminate different types of natural products especially lignans has been relatively well studied [88,89]. Schmidt et al. have analyzed 24 reference lignans including arylnaphthalenes with HPLC- Ultraviolet Detection using a Photodiode Array Detector (HPLC-UV/PAD) and HPLC- Electrospray Ionization/Mass Spectrometry (HPLC-ESI/MS). In comparison to other lignans, the identification of justicidin B as an arylnaphthalene type lignin was easier. Justicidin B showed a strong UV absorbance around 260 nm and a broad absorbance at λ > 300 nm with the stable quasi-molecular ion of [M + H]^+^ = 365 [90]. Other fragments have been summarized in Table 2. Column characteristics and various solvent systems used for the isolation of justicidin B by HPLC and LC-MS in different studies are compared in Table 3, Table 4 and Table 5.

### 7.2. High Speed Counter Current Chromatography (HSCCC) Analysis

Conventional methods for the isolation and purification of secondary metabolites are time consuming and generally results in sample loss. Preparative HSCCC has been reported by Zhou et al. to separate justicidin B with higher recovery from other lignans in *J. procumbens* [91]. Stepwise elution was performed using *n*-hexan:ethylacetate:methanol:water 1.3:1:1.3:1.0, *v*/*v* as solvent system A and 2.5:1.0:2.5:1.0, *v*/*v* as solvent system B. Column temperature was 25 °C, flow rate adjusted at 3 mL/min and rotation speed was 1000 rpm. Justicidin B was eluted after 68 min running of solvent A.

### 7.3. High Performance Thin Layer Chromatography (HPTLC) Analysis

HPTLC analysis has been established as a sensitive and accurate method for the isolation and quantification of justicidin B [92]. R_f_ value for justicidin B was about 0.6 when silica gel TLC plates 60 F_254_ (20 × 10 cm) were scanned by fluorescence at 366 nm after developing in chloroform: methanol 99:1 as the mobile phase.

## 8. Biological Activities

### 8.1. Antifungal Activity

Fungal infections have frightening influence on human health. One of the greatest challenges in fungal therapy is the numerous side effects of antifungal drugs [93]. Justicidin B is a potent inhibitor of *Aspergillus fumigatus*, *Candida albicans* and *Aspergillus flavus* (which are mortal pathogens for immunocompromised patients and cause of women’s disease) with minimum inhibitory concentration (MIC) ≥1, 4 and 12 µg/mL, respectively [10]. Although the potency of justicidin B against, for example, *A. fumigatus* is half of the potency of miconazole or amphotericin B, one should consider the necessity of alternative chemotherapeutics against pathogenic fungi which are resistant to the current medications. *Cryptococcus neoformans* and *Blastoschizomyces capitatus* were both resistant to justicidin B [10]. According to El-Gendy et al. justicidin B shows antifungal activity against *Rhodotorula acuta* (MIC = 3 µg/mL), *Pichia angusta* (MIC = 0.8 µg/mL), *C. neoformans* (MIC = 0.5 µg/mL), *Aspergillus niger* (MIC = 0.2 µg/mL) and *Botrytis fabae* (MIC = 0.4 µg/mL) [41]. However, their experiment was performed in the absence of a positive control.

Comparing the antifungal activity of justicidin B with its precursors against *C. albicans* shows that justicidin B has the strongest anti-*Candida* activity in comparison to (+)-pinoresinol (furofuran-type lignan) [94], lariciresinol (furan-type lignan) [95] and secoisolariciresinol (dibenzylbutan-type lignan) [96]. In Comparison to matairesinol (dibenzylbutyrolactone-type lignan) [97] justicidin B shows strong antifungal activity against *A. niger* and *A. flavus*. After phyllamyricin C (6-methoxyjusticidin B) (MIC = 4 µg/mL), justicidin B is the second strong antifungal agent against *Fusarium oxysporum* (MIC = 8 µg/mL) [98]. There might be a correlation between the antifungal activity of lignans with their structure and lipophilicity. Although, this needs further investigation at least by comparative molecular docking studies or in vitro/in vivo assays.

### 8.2. Antiviral Activity

Mechanism of antiviral activity of lignans is diverse and ranges from tubulin binding to reverse transcriptase inhibition [99]. Justicidin B has displayed strong antiviral activity against vesicular stomatitis virus (VSV) with MIC value about 0.06 µg/mL (MIC < 0.25 µg/mL represents strong antiviral activity) [15]. Justicidin B did not exhibit substantial antiviral activity against human cytomegalovirus [59]. Two hundred seventy four nanomolar (274 nM) justicidin B has shown 74% and 14% of inhibition against Sindbis virus and murine cytomegalovirius (MCMV) grown in mouse 3T3-L1 cells respectively [100,101].

### 8.3. Antibacterial Activity

Contradictory reports exist on the antibacterial activity of justicidin B. Gertsch et al. did not detect any antibacterial effect from justicidin B against *Bacillus cereus*, *Staphylococcus aureus*, *Pseudomonas aeroginosa* and *Escherichia coli* [10]. In a separate study conducted by El-Gendy et al. the MIC value for justicidin B against *E. coli*, *P. aeroginosa*, *Bacillus subtilis*, *B. cereus*, *S. aureus*, *Micrococcus luteus*, *Mycobacterium smegmatis* and *Corynebacterium xerosis* was 0.5, 0.2, 2.0, 2.5, 1.0, 0.5, 5.5 and 7 µg/mL, respectively [41]. No antibiotic was used as the positive control in this study.

### 8.4. Antiparasitic Activity

*Trypanosoma brucei* transmitted by tsetse fly is the cause of trypanosomiasis (sleeping sickness) in African populations [102]. Melarsoprol is the only treatment available for the late stage of disease when the central nervous system (CNS) is invaded [103]. This trivalent arsenical compound is highly toxic, which results in encephalopathy with a 5% death rate in patients [104]. Therefore, there is an urgent need for the discovery of new therapies. Strong activity against the trypomastigote form of *T. brucei rhodesiense* was observed for justicidin B with IC_50_ = 0.2 µg/mL using melarsoprol (IC_50_ = 0.003 µg/mL) as the positive control [10]. Chagas disease the first reason of cardiac mortality in rural areas of Latin America is induced by *Trypanosoma cruzi* [105]. Justicidin B exhibited moderate antiprotozoal activity against *T. cruzi* with IC_50_ = 2.6 µg/mL in comparison to benznidazol (IC_50_ = 0.27 µg/mL) as the positive control [10]. Although lignans from the dibenzylbutyrolactone and dibenzylbutyrolactol subgroups like (−)-hinokinin and (−)-cubebin are stronger trypanocidal agents against *T. cruzi* [106], justicidin B has been considered as one of the strongest anti-trypanosomal lignans towards *T. brucei rhodensis* [107]. Gertsch et al. classified justicidin B as a weak antimalarial agent since this compound had an IC_50_ > 5 µg/mL in comparison to chloroquine (IC_50_ = 0.12 µg/mL) as the positive control [10].

### 8.5. Piscicidal Activity

Piscicides are poisonous chemicals to fish. The piscicidal activity of justicidin B against *Oryzias latipes* fish was comparable with rotenone where no fish survived with a concentration as low as 0.032 ppm after 24 h [108]. The median Tolerance Limit (TLm) after 48 h showed that justicidin B (TLm = 0.04 ppm) is at least 18 times more potent than podophyllotoxin (TLm = 0.73 ppm) [109]. Toxicity of justicidin B was ten times higher than pentachlorophenol (PCP) with the tolerance limit of about 0.028 ppm after 24 h. No significant toxicity of justicidin B against insects like *Musca domestica*, *Periplanata americana*, *Prodenia litura* and *Panonychus citri* has been observed [108]. Ichtyotoxic activity of justicidin B on guppies has been studied by Bachmann et al. [32]. While a concentration of 0.1 ppm affected swimming ability, 1 ppm of justicidin B was lethal [32]. LC_100_ was measured around 1.5 µg/mL after 25–50 min when Gertsch et al. treated adult zebra fish (*Brachydanio rerio*) with justicidin B [10]. This value is about 1 µg/mL for rotenone as the positive control. However, one should consider that in this experiment substances were dissolved in water of basins where justicidin B might not be completely soluble [10]. Therefore, justicidin B could be considered as a strong piscicidal agent.

### 8.6. Antiplatelet Activity

Inhibitors of platelet aggregation play a significant role in the prevention of atherosclerosis and thrombosis development [110]. Several agonists like adrenaline and thromboxane A2 (TxA2) synergize in the activation of platelet aggregation [111]. Cyclooxygenases and thromboxane synthases transform arachidonic acid (AA) to TxA2 [112]. Justicidin B has been tested for its antiplatelet activity using AA to induce platelet aggregation. It is verified that the antiplatelet potency of justicidin B (IC_50_ = 8.0 ± 1 µM) is significantly higher than aspirin (IC_50_ = 20.3 ± 2.1 µM) and less than indomethacin (IC_50_ = 0.21 ± 0.04 µM) [113]. Although adrenaline is a weak adrenergic agonist towards platelet α2-aderenergic receptors, it has a major role in the activation of secondary aggregation. Antiplatelet activity of justicidin B against “adrenalin induced platelet aggregation” for human platelet-rich plasma (PRP) is reported as IC_50_ = 104.8 ± 25.3 µM [114]. Strong antiplatelet effect of justicidin B on AA induced aggregation in comparison to its weak activity on adrenalin induced aggregation could be attributed to the higher binding capacity of plasma for justicidin B. Wu et al. have ascribed antiplatelet activity of justicidin B to the reduction of thromboxane formation [114].

### 8.7. Anti-Inflammatory Activity

Inflammation is involved in various disease states, such as arthritis, atherosclerosis, sepsis, multiple sclerosis and cancer. Several inflammatory mediators including reactive nitrogen oxide (NO) or cytokines like TNF-α and IL-12 are synthesized by macrophages. Justicidin B was able to inhibit LPS-INFγ induced NO and cytokine production from peritoneal murine macrophage with IC_50_ = 12.5 µM [35]. Justicidin B also inhibited NO production up to 50% by pre-activated macrophages with LPS-INFγ for 24 h. Justicidin B inhibits not only the expression of NO synthase gene but also the enzyme activity. Inhibition of LPS-induced NO generation has been studied for other furofuran-type lignans like (+)-piperitol and (+)-sesamine and the dibenzylbutyrolactone-type lignan (−)-hinokinin [115,116]. The inhibitory effect of justicidin B was significantly higher than other derivatives. Rao et al. have compared justicidin B anti-inflammatory activity with diphyllin (7-hydroxyjusticidin B) and phyllamaricin C and concluded that justicidin B has the strongest inhibitory activity [35].

### 8.8. Cytotoxic Activity

Adverse effects and drug resistance to the current onchopharmacologicals have increased the demands for alternative novel therapeutics. In 1984, Pettit et al. showed that justicidin B inhibits the growth of NCI murine P388 lymphocytic leukemia (ED_50_ = 3.3 µg/mL) [30]. An ED_50_ in the range of 1.2 × 10^−2^–7.3 × 10^−2^ µg/mL has been reported for justicidin B towards 9 KB (a subline of HeLa) cell line. LC_50_ of justicidin B on *Artemia salina* (brine shrimp) has been reported to be as much as 1.1 µg/mL [40]. Later, in 1988, Joseph et al. reported weak activity of justicidin B against human bronchial epidermoid carcinoma cell line (NSCLCN6) (Table 6) [16]. Justicidin B displayed low cytotoxic activity against cultured rabbit lung cells (RL-33) with the minimum toxic concentration (MTC) value of about 31 µg/mL (MTC > 31 µg/mL represents a weak cytotoxic agent) [15].

Gertsch et al. determined the cytotoxicity of justicidin B in neoplastic cells (HeLa and Jurkat T cells) and primary cell cultures (L-6 and PBMCs) [10]. Justicidin B displayed nonspecific cytotoxicity in normal and tumor cell lines with the strongest activity against HeLa cells comparable with helenalin as the positive control (Table 6) [10]. Rao et al. reported no cytotoxicity of justicidin B on healthy lines (PBMCs) and negligible cytotoxicity against Jurkat, PC-3, HEPG2 and colon205 cell lines (data for justicidin B has not been reported by Rao et al.) [117].

Although etoposide is widely used in the treatment of leukemia, K562 (chronic myeloid leukemia lines) cells have shown less sensitivity to etoposide [118]. Justicidin B at low doses has shown stronger anti proliferative and propaptotic activity on leukemia cells. Effect of justicidin B on LAMA-84, K-562 and SKW-3 (chronic lymphoid leukemia cell lines) has been studied by Vasilev et al. (Table 6) [69]. LAMA-84 and K-562 cell lines express strongly BCR-ABL oncogene; hence, are less sensitive to chemotherapeutics. All three cell lines were sensitive to justicidin B. However, justicidin B was less active than etoposide to some degree especially against K-562. Justicidin B activates programmed cell death since the authors observed DNA fragmentation after treatment of cells with justicidin B. Luo et al. have investigated the detailed effect of justicidin B on human leukemia K562 cells [119]. Using MTT assay IC_50_ of justicidin B after 48 h was 45.4 µM on K562 cells, which is at least seven times higher than Vasilev et al’s. report. Effect of justicidin B on L1210 and P388D1 cell lines after 48 h has shown that justicidin B has higher activity than etoposide towards L1210 cells (Table 6).

Superoxide dismutase (SOD) partitionates the superoxide (O_2_^−^) radical to protect cells from peroxidation injury. Rao et al. could not detect any significant scavenging activity of justicidin B against superoxide anion with concentrations up to 100 µM [117]. Justicidin B significantly decreased the activity of SOD in a dose dependent manner in a separate study. 3, 11.9 and 47.6 µM of justicidin B decreased SOD activity 46.4%, 52.6% and 84.3%, respectively. Justicidin B manipulates redox homeostasis in K562 leukemia cells by the deterioration of ROS deletion [119]. It has been demonstrated that contrary to podophyllotoxin which increases the proportion of K562 cells at metaphase, justicidin B arrests the cell development at subG0 phase [119]. The percentage of apoptosis rate for 67.6 µM justicidin B was 14.6% ± 1.3% for K562 cells. Cleavage of some cellular proteins by Caspase 3 results in apoptosis. 8.4, 16.9 and 67.6 µM of justicidin B increased caspase 3 activity 11.2%, 13.9% and 19.1%, respectively [119], which indicates that justicidin B induces apoptosis in K562 cells via caspase-dependent pathway.

Ctotoxicity of justicidin B against MDA-MB-231 and MCF-7 ER-negative and ER positive breast carcinoma cell lines has been determined [120]. Justicidin B exhibited stronger activity against MCF-7 than MDA-MB-231. MCF-7 was more sensitive to justicidin B than to etoposide (control) (Table 6). Momekov et al. have attributed the observed cytotoxicity to the induced DNA fragmentation by justicidin B [120]. Since caspase inhibitors like Boc-D-FMK has abolished proapoptosis when cells were co-incubated with both justicidin B and Boc-D-FMK, one can conclude that justicidin B triggers programmed cell death through caspase mechanism. As ER-positive cell line showed more sensitivity to the treatment, it was concluded that justicidin B might act through estrogen receptor regulation. Additionally, the treatment of MDA-MB-231 with justicidin B decreased Nuclear Factor-Kappa B (NF-κB) expression in a similar manner to etoposide.

Momekov et al. evaluated onchopharmacological characteristics of justicidin B in the acute myeloid leukemia derived cell line HL-60 in the absence of a positive control [121]. Justicidin B IC_50_ value was about 3.6 ± 0.07 µM after 24 h treatment with a significant increase in apoptotic DNA fragmentation. They have concluded that justicidin B activates the intrinsic mitochondrial cell death signaling pathway via caspase 3 and caspase 9 [121].

Justicidin B has also shown potential cytotoxicity against human L0V0 colorectal carcinoma and human gastric cancer cell line (BGC-823) [122]. Diphyllin (7-hydroxyjusticidin B) has been used as the positive control in this study. Stronger cytotoxicity of justicidin B against BGC-823 cell line has been attributed to the lower polarity of justicidin B [122]. Effect of justicidin B on eight different lymphoma derived cell lines has been assessed by Ilieva et al. [123]. The effect of justicidin B on lymphoma cell lines is summarized in Table 7. After 72 h of incubation multiple myeloma (MM) cell lines, MM (RPMI-8226) and MM (OPM2) were the most sensitive cell lines in the presence of justicidin B. Sensitivity of RPMI-8226 was higher in the presence of justicidin B than etoposide. DOHH-2 (human non-Hodgkin lymphoma (NHL) cell line), HuT-78 (cutaneous T cell lymphoma) and REH (human B cell precursor leukemia) were also sensitive to justicidin B. However, etoposide was a slightly stronger cytotoxic agent for these three cell lines. Hodgkin’s lymphoma cell line (HD-MY-Z) did not show any sensitivity towards justicidin B and etoposide.

The transcription factor NF-κB involves in the regulation of many genes that code for mediators of apoptosis and tumorigenesis. NF-κB is composed of homo- and heterodimeric complexes of the Rel family containing p50, p65 (RelA), c-Rel, p52 and RelB. The best-characterized form of NF-κB is the p65/p50 heterodimer [124]. In chronic inflammation and malignancies, p65 is constitutively active. Therefore, inhibitors of p65 are important drug targets to control malignancies. Justicidin B inhibits the expression of NF-κB in human NHL (DOHH-2) and CTCL (HH) cell lines. In DOHH-2 and RPMI-8226, caspase 8, which initiates TNF-induced apoptosis is activated by justicidin B. However, NF-κB expression is induced by TNF, this induction counteracts NF-κB inhibition. In DOHH-2 cell lines, procaspase 3 was also decreased (caspase 3 activation). Although caspase 8 activity was increased in RPMI-8226 cells, no change in procaspase 3 and NF-κB was observed suggesting the involvement of other mechanisms of cell death in these lines [123].

### 8.9. Evaluation of in Vivo Animal Toxicity

No in vivo toxicity has yet been reported for justicidin B. Justicidin B was injected intraperitoneally (i.p.) to H-albino mice by Ilieva et al. [123]. A maximum dose of 50 mg/kg (137.25 µM) justicidin B resulted in no physical or behavioral differences. No changes were observed in food and water consumption or mortality rate after two weeks.

### 8.10. Miscellaneous Activities

Bone resorption inhibitors are therapeutic agents for patients with excess calcium in the blood or bone cancer. In 1996, justicidin B was introduced as a bone resorption inhibitor [125]. Twenty five micromolar (25 µM) of justicidin B could inhibit Ca release from bone up to 47%, which might be useful in osteoclastogenesis and cancer.

The effect of justicidin B on the detoxification of pollutants has been also tested [126]. Incomplete combustion at high temperatures results in the formation of benzo[a]pyrene (BaP) which after hydroxylation by CYP1A1 enzyme in the liver forms highly carcinogenic metabolites [127]. Inhibitors of BaP hydroxylase can potentially decrease the danger of mutagenicity [128]. Ten micromolar (10 µM) justicidin B could decrease BaP hydroxylase by 21% [126].

Free radical reactions cause damage to macromolecules and results in various disease states. Antioxidant properties of justicidin B has been tested against DPPH radical. Justicidin B displayed moderate scavenging activity with IC_50_ = 65.0 ± 1.8 µM [117].

## 9. Conclusions

A wide array of biological activities has been ascribed to justicidin B. The cytotoxic, antifungal and antiprotozoal effects demonstrate that justicidin B plays a major role as the chemical defense machinery of the corresponding species. Pharmaceutically valuable characteristics of justicidin B (especially the cytotoxic properties) resulted in considerable interest toward its production. Multiple myeloma as a debilitating malignancy is usually refractory and multidrug resistance. The annual treatment of leukemia costs more than $2 billion only in USA still more than half of the patients expire. Cytotoxic effect of justicidin B on 23 different cell lines has been compared. In comparison to the aryltetralin lignans, which are currently used for the treatment of malignancies, the strongest activity of justicidin B was against L1210 the lymphocytic leukemia cells and RPMI8226 the multiple myeloma cell line. This provides insights for introduction of a new compound into the clinical trial studies as a replacement or additive therapy. In addition to the specific cytotoxicity of justicidin B for L1210 and RPMI8226 cells, antifungal activity against *C. albicans*, *A. niger*, *A. flavus* and *F. oxysporum* are distinguished properties of justicidin B. Antiprotozal effects of justicididin B against trypomastigote forms of *T. brucei rhodensis* is one of the most prominent activities of justicidin B over other lignans. In comparison to several other derivatives, justicidin B showed a dominant anti-inflammatory activity. Stronger anti-platelet effects of justicidin B in comparison to aspirin might be beneficial for the patients to whom aspirin is either contraindicated or does not result in therapeutic effects.

In this review, we focused on the in vivo and in vitro distribution of justicidin B in plant families of Acanthaceae, Euphorbiaceae, Linaceae and Rutaceae. Reliable, robust and selective analysis of justicidin B by chromatographic methods was described. Reports which have been published featuring short and efficient total synthesis of this interesting natural product focusing on the construction of naphthalene ring as the key step in the synthetic strategy were compared. Cationic cyclization and intramolecular Diels-Alder reaction were the most affordable synthetic strategies. Difficulties in the total synthesis of justicidin B reveal how crucial is to establish and screen in vitro cell, tissue and organ cultures to optimize justicidin B production. Elucidation of the unknown enzymes and encoding genes in the biosynthetic pathway of justicidin B provides the opportunity to genetically manipulate and improve plants, producing high yields of the corresponding metabolite, which is economically feasible for scale up purposes.

## Figures and Tables

**Table 1:** Detailed NMR characteristics of justicidin B in CDCl_3_ [9].

C	δ_C_ (ppm)	H	δ_H_ (ppm)	Multiplicity	Coupling Constant (Hz)	gHMBC	gCOSY	gHSQC
1′	128.3	-	-	-	-	-	-	-
2′	110.5	2′	6.78	d	*J* (2′, 6′) = 1.5	C-3′, 6′, 1′, 8′	H-5′, 6′, 12	H-2′
3′	148.0	-	-	-	-	-	-	-
4′	147.8	-	-	-	-	-	-	-
5′	105.8	5′	6.98	d	*J* (5′, 6′) = 7.8	C-6′, 1′, 4′	H-2′, 6′	H-5′
6′	123.4	6′	6.77	d	*J* (6′, 5′) = 7.8 *J* (6′, 2′) = 1.5	C-4′, 8′, 2′, 5′	H-5′, 2′	H-6′
7′	139.5	-	-	-	-	-	-	-
8′	118.4	-	-	-	-	-	-	-
9′	169.9	-	-	-	-	-	-	-
10	101.2	10a 10b	6.05	s	-	C-4′, 3′	H-2′	H-10a H-10b
1	133.1	-	-	-	-			
2	128.8	-	-	-	-	-	-	-
3	108.1	5	7.08	s	-	C-7, 2, 1	H-6, 7, 11	H-3
4	151.7	-	-	-	-	-	-	-
5	150.0	-	-	-	-	-	-	-
6	106.0	2	7.12	s	-	C-5, 1, 2, 7	H-7, 10	H-6
7	118.2	7	7.61	s	-	C-2, 8′, 6	H-9, 6, 3	H-7
8	139.4	-	-	-	-	-	-	-
9	67.9	9a 9b	5.33	s	-	C-8′, 2, 1, 8, 9′	H-7	H-9a H-9b
11	56.0	11	3.70	s	-	C-5	H-7	H-11
12	55.8	12	3.95	s	-	C-4	H-3	H-12

**Table 2:** ESI/MS fragmentation of justicidin B [90].

Molecular Mass	364			
Cluster ions	[2M + Na]^+^	[2M + NH_4_]^+^	[2M + H]^+^	
	751 ^c^	746 ^a^	-	
Quasi molecular ions	[M + Na]^+^	[M + NH_4_]^+^	[M + H]^+^	
	387 ^a^	-	365 ^d^	
Fragment ions	[M + H − H_2_O]^+^	[M + H − CH_2_O]^+^	[M + H − CO_2_]^+^	
	-	335 ^c^	321 ^c^	303 ^b^ 291 ^b^ 289 ^b^

Relative ion intensities: ^a^ detectable, <1%; ^b^ 1%–10%; ^c^ 11%–20%; ^d^ 100%.

**Table 3:** Conditions to isolate and identify justicidin B by HPLC.

Column	A (Solvent)	B (Solvent)	Time (min)	Flow Rate (mL/min)	A%	B%	Rt * (min)	Ref.
YMC pack ODS-A (150 mm × 4.6 mm × 5 µm) (25 °C)	water	methanol	0	0.8	90	10	49	[91]
			60		10	90		
			70		10	90		
Nucleosil 100-C_18_ (40 + 250 mm × 4.6 mm × 5 µm)	water	acetonitrile	0	0.8	55	45	15.51	[8] **
			17		33	67		
			18		50	50		
			25		55	45		
Nucleodur 100-5 C_18_ (125 mm × 4.6 mm × 5 µm)	water	methanol	0	1.4	70	30	26.5	[90]
			30		30	70		
			45		0	100		
			55		0	100		
GROM-Sil C_18_ (20 + 250 mm × 4 mm × 5 µm)	(0.1% H_3_PO_4_) in water	acetonitrile	0	0.8	75	25	-	[74]
			25	0.8	62	38		
			43	1	57	43		
			46	1	45	55		
			54	1	30	70		
			56	1	75	25		
			60	0.8	75	25		
Shiseido Capcell Pak C_18_ (250 mm × 4.6 mm × 5 µm) (35 °C)	water	acetonitrile	0	0.8	80	20	54	[11]
			40		54	46		
			60		45	55		
			70		40	60		
			75		60	40		

* Rt = Retention time; ** Similar conditions using a column with 8.0 mm i.d. and a flow rate of 3 mL/min between 0 and 11 min, 0.8 mL/min from 11 to 18 min back to 3 mL/min between 18 and 25 min was used for the isolation of justicidin B by semipreparative HPLC.

**Table 4:** Conditions to isolate and identify justicidin B by HPLC-ESI/MS [90].

Parameters	Conditions		
HPLC-ESI/MS			
**Column: Knauer Eurosphere RPC18 (250 mm × 2 mm × 5 µm)**			
	**Time (min)**	**A% (Water + 0.1% Formic Acid)**	**B% (Acetonitrile)**
Gradient	0	70	30
	30	30	70
	40	30	70
	55	0	100
	65	0	100
Flow rate	0.4 mL/min		
Ionization mode	ESI + and − mode		
Capillary temp	300 °C		
Source voltage	5 kV		

**Table 5:** Conditions to isolate and identify justicidin B by HPLC-ESI/MS^n^ [11].

Parameters	Conditions		
HPLC-ESI/MS^n^			
**Column: Shiseido Capcell Pak C_18_ (250 mm × 4.6 mm × 5 µm) (35 °C)**			
	**Time (min)**	**A% (Water)**	**B% (Acetonitrile)**
Gradient	0	80	20
	40	54	46
	60	45	55
	70	60	40
	75	60	40
Flow rate	0.8 mL/min		
Ionization mode	ESI + mode		
Capillary temp	330 °C		
Source voltage	4.5 kV		
Capillary voltage	18 V		
Sheath gas	(N_2_) flow, 50 AU		
Auxillary gas	(N_2_) flow, 15 AU		

**Table 6:** Cytotoxicity of justicidin B against selected tumor cell lines.

Cell Line	Justicidin B	Diphyllin	Etoposide	Helanin
NSCLCN6	28 *	-	-	-
KB HeLa	0.2 *	-	-	0.2 *
Jurkat T	3.2 *	-	-	0.03 *
L6	3.3 *	-	-	-
PBM Cs	4.7 *	-	-	0.03 *
LAMA-84	1.11	-	0.79	-
K-562	6.08	-	1.87	-
SKW-3	1.62	-	0.82	-
MDA-MB-231	106.9	-	63.1	-
MCF-7	38.7	-	52.7	-
HL-60	0.9	-	-	-
L1210	6.3	-	14.2	-
P388D1	7.3	-	-	-
L0V0	6.081	8.120	-	-
BGC-823	0.179	8.079	-	-

Units indicate IC_50_ in µM. Numbers marked by asterisks (*) are µg/mL.

**Table 7:** Cytotoxiccity of justicidin B and etoposide (positive control) on lymphoma derived cell lines after 72 h exposure.

Compound	HD-MY-Z	DOHH-2	REH	HH	HuT-78	OPM-2	RPMI-8226	U-266
justicidin B	144.5	5	8	16.2	6.1	1.5	0.17	17.2
etoposide	>100	9.5	0.015	14.7	4.2	1.3	14.9	27.4

Units indicate IC_50_ in µM.

## References

[1] Davin L.B., Lewis N.G. (2003). An historical perspective on lignan biosynthesis: Monolignol, allylphenol and hydroxycinnamic acid coupling and downstream metabolism. Phytochem. Rev..

[2] Umezawa T. (2003). Diversity in lignan biosynthesis. Phytochem. Rev..

[3] Lee K.H., Xiao Z. (2003). Lignans in treatment of cancer and other diseases. Phytochem. Rev..

[4] Canel C., Moraes R.M., Dayan F.E., Ferreira D. (2000). Podophyllotoxin. Phytochemistry.

[5] DellaGreca M., Zuppolini S., Zarrelli A. (2013). Isolation of lignans as seed germination and plant growth inhibitors from mediterranean plants and chemical synthesis of some analogues. Phytochem. Rev..

[6] Charlton J.L., Oleschuk C.J., Chee G.L. (1996). Hindered rotation in arylnaphthalene lignans. J. Org. Chem..

[7] Okigawa M., Maeda T., Kawano N. (1970). The isolation and structure of three new lignans from *Justicia procumbens* linn. var. *Leucantha honda*. Tetrahedron.

[8] Mohagheghzadeh A., Schmidt T.J., Alfermann A.W. (2002). Arylnaphthalene lignans from in vitro cultures of *Linum austriacum*. J. Nat. Prod..

[9] Da Silva R., Ruas M.M., Donate P.M. (2007). Complete assignments of ^1^H and ^13^C NMR spectral data for arylnaphthalene lignana lactones. Magn. Reson. Chem..

[10] Gertsch J., Tobler R.T., Brun R., Sticher O., Heilmann J. (2003). Antifungal, antiprotozoal, cytotoxic and piscicidal properties of justicidin B and a new aryinaphthalide lignan from *Phyllanthus piscatorum*. Planta Med..

[11] Wang L., Pan J., Yang M., Wu J., Yang J. (2011). Chromatographic fingerprint analysis and simultaneous determination of eight lignans in *Justicia procumbens* and its compound preparation by HPLC-DAD. J. Sep. Sci..

[12] Munakata K., Marumo S., Ohta K., Chen Y.L. (1965). Justicidin A and B, the fish-killing components of *Justicia hayatai* var. *Decumbens*. Tetrahedron Lett..

[13] Govindachari T.R., Sathe S.S., Viswanathan N., Pai B.R., Srinivasan M. (1967). Revised structures of diphyllin and justicidin A. Tetrahedron Lett..

[14] Corrêa G.M., de Minas C. (2012). ; Federal University of Minas Gerais. Chemical constituents and biological activities of species of *Justicia*: A review. Rev. Bras. Farmacogn..

[15] Asano J., Chiba K., Tada M., Yoshii T. (1996). Antiviral activity of lignans and their glycosides from *Justicia procumbens*. Phytochemistry.

[16] Joseph H., Gleye J., Moulis C., Mensah L.J., Roussakis C., Gratas C. (1988). Justicidin B, a cytotoxic principle from *Justicia pectoralis*. J. Nat. Prod..

[17] Day S.H., Chiu N.Y., Won S.J., Lin C.N. (1999). Cytotoxic lignans of *Justicia ciliata*. J. Nat. Prod..

[18] Kavitha J., Gopalaiah K., Rajasekhar D., Subbaraju G.V. (2003). Juspurpurin, an unusual secolignan glycoside from *Justicia purpurea*. J. Nat. Prod..

[19] Ulubelen A., Öztürk M. (2008). Alkaloids, coumarins and lignans from *Haplophyllum* species. Rec. Nat. Prod..

[20] Batirov E.K., Matkarimov A.D., Malikov V.M. (1981). Justicidin B and diphyllin from *Haplophyllum obtusifolium*. Khimija Prir. Soyedineniy.

[21] Abdullaev N.D., Yagudaev M.R., Batirov E.K., Malikov V.M. (1987). ^13^C NMR spectra of arylnaphthalene lignans. Chem. Nat. Compd..

[22] Batsuren D., Batirov E.K., Malikov V.M., Zemlyanskii V.N., Yagudaev M.R. (1982). Arylnaphthalene lignans of *Haplophyllum dauricum*. The structure of daurinol. Chem. Nat. Compd..

[23] Ulubelen A. (1985). Alkaloids from *Haplophyllum buxbaumii*. Phytochemistry.

[24] Nukul G.S., Abu Zarga M.H., Sabri S.S., Al-Eisawi D.M. (1987). Chemical constituents of the flora of Jordan, part III. Mono-*o*-acetyl diphyllin apioside, a new arylnaphthalene lignan from *Haplophyllum buxbaumii*. J. Nat. Prod..

[25] Sheriha G.M., Abou Amer K.M. (1984). Lignans of *Haplophyllum tuberculatum*. Phytochemistry.

[26] Al-Yahya M.A., El-Domiaty M.M., Al-Meshal I.A., Al-Said M.S., El-Feraly F.S. (1991). (+)-Dihydroperfamine: An alkaloid from *Haplophyllum tuberculatum*. Int. J. Pharmacogn..

[27] Gozler B., Arar G., Gozler T., Hesse M. (1992). Isodaurinol, an arylnaphthalene lignan from *Haplophyllum cappadocicum*. Phytochemistry.

[28] Puricelli L., Innocenti G., Piacente S., Caniato R., Filippini R., Capelletti E.M. (2002). Production of lignans by *Haplophyllum patavinum* in vivo and in vitro. Heterocycles.

[29] Qi W., Hua L., Gao K. (2014). Chemical constituents of the plants from the genus *Phyllanthus*. Chem. Biodivers..

[30] Pettit G.R., Cragg G.M., Suffness M.I., Gust D., Boettner F.E., Williams M., Saenz-Renauld J.A., Brown P., Schmidt J.M., Ellis P.D. (1984). Antineoplastic agents. 104. Isolation and structure of the *Phyllanthus acuminatus* vahl (Euphorbiaceae) glycosides. J. Org. Chem..

[31] Pettit G.R., Schaufelberger D.E. (1988). Isolation and structure of the cytostatic lignan glycoside phyllanthostatin A. J. Nat. Prod..

[32] Bachmann T.L., Ghia F., Torssell K.B.G. (1993). Lignans and lactones from *Phyllanthus anisolobus*. Phytochemistry.

[33] Lin M.-T., Lee S.-S., Chen Liu K.C.S. (1995). Phyllamyricins A-C, three novel lignans from *Phyllanthus myrtifolius*. J. Nat. Prod..

[34] Wang C.Y., Lam S.H., Tseng L.H., Lee S.S. (2011). Rapid screening of lignans from *Phyllanthus myrtifolius* and stilbenoids from *Syagrus romanzoffiana* by HPLC-SPE-NMR. Phytochem. Anal..

[35] Rao Y.K., Fang S.H., Tzeng Y.M. (2006). Anti-inflammatory activities of constituents isolated from *Phyllanthus polyphyllus*. J. Ethnopharmacol..

[36] Schmidt T.J., Hemmati S., Klaes M., Konuklugil B., Mohagheghzadeh A., Ionkova I., Fuss E., Alfermann A.W. (2010). Lignans in flowering aerial parts of *Linum* species—Chemodiversity in the light of systematics and phylogeny. Phytochemistry.

[37] Schmidt T.J., Vossing S., Klaes M., Grimme S. (2007). An aryldihydronaphthalene lignan with a novel type of ring system and further new lignans cells from *Linum perenne* L.. Planta Med..

[38] Vasilev N., Ebel R., Edrada R., Fuss E., Alfermann A.W., Ionkova I., Petrova A., Repplinger M., Schmidt T.J. (2008). Metabolic profiling of lignan variability in *Linum* species of section Syllinum native to Bulgaria. Planta Med..

[39] Schmidt T.J., Klaes M., Sendker J. (2012). Lignans in seeds of *Linum* species. Phytochemistry.

[40] Hui Y.H., Chang C.J., McLaughlin J.L., Powell R.G. (1986). Justicidin B, a bioactive trace lignan from the seeds of *Sesbania drummondii*. J. Nat. Prod..

[41] El-Gendy M.M.A., Hawas U.W., Jaspars M. (2008). Novel bioactive metabolites from a marine derived bacterium *Nocardia* sp. ALAA 2000. J. Antibiot..

[42] Munakata K., Marumo S., Ohta K., Chen Y.L. (1967). The synthesis of justicidin B and related compounds. Tetrahedron Lett..

[43] Block E., Stevenso R. (1971). Lignan lactones. Synthesis of (±)-collinusin and justicidin B. J. Org. Chem..

[44] Momose T., Kanai K., Hayashi K. (1978). Synthetic studies on lignans and related compounds. VIII. Synthesis of justicidin B and diphyllin and of taiwanin C and E from 2,3-dibenzylidenebutyrolactones via β-apolignans. Chem. Pharm. Bull..

[45] Ghosal S., Banerjee S. (1979). Synthesis of retrochinensin; a new naturally occurring 4-aryl-2,3-naphthalide lignan. J. Chem. Soc. Chem. Commun..

[46] Rodrigo R. (1988). Progress in the chemistry of isobenzofurans: Applications to the synthesis of natural products and polyaromatic hydrocarbons. Tetrahedron.

[47] Ogiku T., Seki M., Takahashi M., Ohmizu H., Iwasaki T. (1990). A new two-step synthesis of 1-arylnaphthalene lignans from cyanohydrins. Tetrahedron Lett..

[48] Kamal A., Daneshtalab M., Micetich R.G. (1994). A rapid entry into podophyllotoxin congeners: Synthesis of justicidin B. Tetrahedron Lett..

[49] Kobayashi K., Tokimatsu J., Maeda K., Morikawa O., Konishi H. (1995). New, short synthesis of arylnaphthofuranone lignans based on reactions of *o*-aroylbenzyllithiums with furan-2(5*H*)-one. J. Chem. Soc. Perkin Trans. 1.

[50] Cochran J.E., Padwa A. (1995). A new approach to the 1-arylnaphthalene lignans utilizing a tandem Pummerer-Diels-Alder reaction sequence. J. Org. Chem..

[51] Harrowven D.C., Bradley M., Castro J.L., Flanagan S.R. (2001). Total syntheses of justicidin B and retrojusticidin B using a tandem Horner-Emmons-Claisen condensation sequence. Tetrahedron Lett..

[52] Flanagan S.R., Harrowven D.C., Bradley M. (2002). A new benzannulation reaction and its application in the multiple parallel synthesis of arylnaphthalene lignans. Tetrahedron.

[53] Nishii Y., Yoshida T., Asano H., Wakasugi K., Morita J., Aso Y., Yoshida E., Motoyoshiya J., Aoyama H., Tanabe Y. (2005). Regiocontrolled benzannulation of diaryl (*gem*-dichlorocyclopropyl) methanols for the synthesis of unsymmetrically substituted α-arylnaphthalenes: Application to total synthesis of natural lignan lactones. J. Org. Chem..

[54] Foley P., Eghbali N., Anastas P.T. (2010). Silver-catalyzed one-pot synthesis of arylnaphthalene lactone natural products. J. Nat. Prod..

[55] Mondal S., Maji M., Basak A. (2011). A Garratt-Braverman route to aryl naphthalene lignans. Tetrahedron Lett..

[56] Patel R.M., Argade N.P. (2013). Palladium-promoted [2+2+2] cocyclization of arynes and unsymmetrical conjugated dienes: Synthesis of justicidin B and retrojusticidin B. Org. Lett..

[57] Kudoh T., Shishido A., Ikeda K., Saito S., Ishikawa T. (2013). Concise synthesis of arylnaphthalene lignans by regioselective intramolecular anionic Diels-Alder reactions of 1,7-diaryl-1,6-diynes. Synlett.

[58] Kocsis L.S., Brummond K.M. (2014). Intramolecular dehydro-Diels-Alder reaction affords selective entry to arylnaphthalene or aryldihydronaphthalene lignans. Org. Lett..

[59] Cow C., Leung C., Charlton J.L. (2000). Antiviral activity of arylnaphthalene and aryldihydronaphthalene lignans. Can. J. Chem. Rev. Can. Chim..

[60] Wink M., Alfermann A.W., Franke R., Wetterauer B., Distl M., Windhövel J., Krohn O., Fuss E., Garden H., Mohagheghzadeh A. (2005). Sustainable bioproduction of phytochemicals by plant in vitro cultures: Anticancer agents. Plant Genet. Resour. C.

[61] Ionkova I., Arora R. (2010). Podophyllotoxin and related lignans: Biotechnological production by in vitro plant cell cultures. Medicinal Plant Biotechnology.

[62] Fuss E. (2003). Lignans in plant cell and organ cultures: An overview. Phytochem. Rev..

[63] Alfermann A., Petersen M., Fuss E., Laimer M., Rucker W. (2003). Production of natural products by plant cell biotechnology: Results, problems and perspectives. Plant Tissue Culture 100 Years since Gottlieb Haberlandt.

[64] Innocenti G., Puricelli L., Piacente S., Caniato R., Filippini R., Cappelletti E.M. (2002). Patavine, a new arylnaphthalene lignan glycoside from shoot cultures of *Haplophyllum patavinum*. Chem. Pharm. Bull..

[65] Konuklugil B., Ionkova I., Vasilev N., Schmidt T.J., Windhövel J., Fuss E., Alfermann A.W. (2007). Lignans form *Linum* species of sections Syllinum and Linum. Nat. Prod. Res..

[66] Nikolov S., Momekov G., Kitanov G., Ionkova I., Krasteva I., Toshkova R., Konstantinov S., Nedialkov P., Karaivanova M. (2007). Exploitation of the bulgarian flora's biodiversity as a source of immunomodulatory and/or antineoplastic agents: Current challenges and perspectives. Biotechnol. Biotechnol. Equip..

[67] Vasilev N.P., Ionkova I. (2005). Cytotoxic activity of extracts from *Linum* cell cultures. Fitoterapia.

[68] Ionkova I., Sasheva P., Ionkov T., Momekov G. (2013). *Linum narbonense*: A new valuable tool for biotechnological production of a potent anticancer lignan justicidine B. Pharmacogn. Mag..

[69] Vasilev N., Elfahmi, Bos R., Kayser O., Momekov G., Konstantinov S., Ionkova I. (2006). Production of justicidin B, a cytotoxic arylnaphthalene lignan from genetically transformed root cultures of *Linum leonii*. J. Nat. Prod..

[70] Ionkova I., Sasheva P., Momekov G. (2011). Justicidin B—A potent cytotoxic arylnaphtalene lignan from in vitro cultures of *Linum leonii*. Planta Med..

[71] Vasilev N., Ionkova I. (2005). Lignan production by cell cultures of *Linum setaceum* and *Linum campanulatum*. Pharm. Biol..

[72] Mohagheghzadeh A., Dehshahri S., Hemmati S. (2009). Accumulation of lignans by in vitro cultures of three *Linum* species. Z. Naturforsch. C.

[73] Hemmati S., Schneider B., Schmidt T., Federolf K., Alfermann A.W., Fuss E., Skaltsounis L., Magiatis P. Biosynthesis of justicidin B and diphyllin in cell cultures of *Linum perenne* L. Himmelszelt. Proceedings of the 7th Joint Meeting of GA, AFERP, ASP, ESP & SIF.

[74] Hemmati S., Schmidt T.J., Fuss E. (2007). (+)-Pinoresinol/(−)-lariciresinol reductase from *Linum perenne* Himmelszelt involved in the biosynthesis of justicidin B. FEBS Lett..

[75] Jullian-Pawlicki N., Lequart-Pillon M., Huynh-Cong L., Lesur D., Cailleu D., Mesnard F., Laberche J.C., Gontier E., Boitel-Conti M. (2015). Arylnaphthalene and aryltetralin-type lignans in hairy root cultures of *Linum perenne*, and the stereochemistry of 6-methoxypodophyllotoxin and one diastereoisomer by HPLC-MS and NMR spectroscopy. Phytochem. Anal..

[76] Satake H., Koyama T., Bahabadi S.E., Matsumoto E., Ono E., Murata J. (2015). Essences in metabolic engineering of lignan biosynthesis. Metabolites.

[77] Hemmati S., Schneider B., Schmidt T.J., Federolf K., Alfermann A.W., Fuss E. (2007). Justicidin B 7-hydroxylase, a cytochrome p450 monooxygenase from cell cultures of *Linum perenne* Himmelszelt involved in the biosynthesis of diphyllin. Phytochemistry.

[78] Davin L.B., Bedgar D.L., Katayama T., Lewis N.G. (1992). On the stereoselective synthesis of (+)-pinoresinol in *Forsythia suspensa* from its achiral precursor, coniferyl alcohol. Phytochemistry.

[79] Halls S.C., Davin L.B., Kramer D.M., Lewis N.G. (2004). Kinetic study of coniferyl alcohol radical binding to the (+)-pinoresinol forming dirigent protein. Biochemistry.

[80] Pickel B., Constantin M.A., Pfannstiel J., Conrad J., Beifuss U., Schaller A. (2010). N enantiocomplementary dirigent protein for the enantioselective lacease-catalyzed oxidative coupling of phenols. Angew. Chem. Int. Ed..

[81] Davin L.B., Wang H.B., Crowell A.L., Bedgar D.L., Martin D.M., Sarkanen S., Lewis N.G. (1997). Stereoselective bimolecular phenoxy radical coupling by an auxiliary (dirigent) protein without an active center. Science.

[82] Kazenwadel C., Klebensberger J., Richter S., Pfannstiel J., Gerken U., Pickel B., Schaller A., Hauer B. (2013). Optimized expression of the dirigent protein *At*DIR6 in *Pichia pastoris* and impact of glycosylation on protein structure and function. Appl. Microbiol. Biotechnol..

[83] Kim K.W., Moinuddin S.G.A., Atwell K.M., Costa M.A., Davin L.B., Lewis N.G. (2012). Opposite stereoselectivities of dirigent proteins in arabidopsis and schizandra species. J. Biol. Chem..

[84] Hemmati S., von Heimendahl C.B., Klaes M., Alfermann A.W., Schmidt T.J., Fuss E. (2010). Pinoresinol-lariciresinol reductases with opposite enantiospecificity determine the enantiomeric composition of lignans in the different organs of *Linum usitatissimum* L.. Planta Med..

[85] Fujita M., Gang D.R., Davin L.B., Lewis N.G. (1999). Recombinant pinoresinol-lariciresinol reductases from western red cedar (*Thuja plicata*) catalyze opposite enantiospecific conversions. J. Biol. Chem..

[86] Xia Z.Q., Costa M.A., Pelissier H.C., Davin L.B., Lewis N.G. (2001). Secoisolariciresinol dehydrogenase purification, cloning, and functional expression. Implications for human health protection. J. Biol. Chem..

[87] Wolfender J.L. (2009). HPLC in natural product analysis: The detection issue. Planta Med..

[88] Liu X.T., Wang X.G., Xu R., Meng F.H., Yu N.J., Zhao Y.M. (2015). Qualitative and quantitative analysis of lignan constituents in *Caulis trachelospermi* by HPLC-QTOF-MS and HPLC-UV. Molecules.

[89] Eklund P.C., Backman M.J., Kronberg L.Å., Smeds A.I., Sjöholm R.E. (2008). Identification of lignans by liquid chromatography-electrospray ionization ion-trap mass spectrometry. J. Mass Spectrom..

[90] Schmidt T.J., Hemmati S., Fuss E., Alfermann A.W. (2006). A combined HPLC-UV and HPLC-MS method for the identification of lignans and its application to the lignans of *Linum usitatissimum* L. and *L. bienne* Mill. Phytochem. Anal..

[91] Zhou P., Luo Q., Ding L., Fang F., Yuan Y., Chen J., Zhang J., Jin H., He S. (2015). Preparative isolation and purification of lignans from *Justicia procumbens* using high-speed counter-current chromatography in stepwise elution mode. Molecules.

[92] Vasilev N., Nedialkov P., Ionkova I., Ninov S. (2004). HPTLC densitomeric determination of justicidin B in *Linum* in vitro cultures. Pharmazie.

[93] Brown G.D., Denning D.W., Gow N.A., Levitz S.M., Netea M.G., White T.C. (2012). Hidden killers: Human fungal infections. Sci. Transl. Med..

[94] Hwang B., Lee J., Liu Q.H., Woo E.R., Lee D.G. (2010). Antifungal effect of (+)-pinoresinol isolated from *Sambucus williamsii*. Molecules.

[95] Hwang B., Cho J., Hwang I.S., Jin H.G., Woo E.R., Lee D.G. (2011). Antifungal activity of lariciresinol derived from *Sambucus williamsii* and their membrane-active mechanisms in *Candida albicans*. Biochem. Biophys. Res. Commun..

[96] Välimaa A.L., Honkalampi-Hämäläinen U., Pietarinen S., Willför S., Holmbom B., von Wright A. (2007). Antimicrobial and cytotoxic knotwood extracts and related pure compounds and their effects on food-associated microorganisms. Int. J. Food Microbiol..

[97] Panagouleas C., Skaltsa H., Lazari D., Skaltsounis A.L., Sokovic M. (2003). Antifungal activity of secondary metabolites of *Centaurea raphanina* ssp. *mixta*, growing wild in greece. Pharm. Biol..

[98] Windayani N., Rukayadi Y., Hakim E.H., Ruslan K., Syah Y.M. (2014). Antifungal activity of lignans isolated from *Phyllanthus myrtifolius* Moon. against *Fusarium oxysporum*. Phytochemistry.

[99] Chang C.W., Lin M.T., Lee S.S., Liu K.C., Hsu F.L., Lin J.Y. (1995). Differential inhibition of reverse transcriptase and cellular DNA polymerase-alpha activities by lignans isolated from chinese herbs, *Phyllanthus myrtifolius* moon, and tannins from *Lonicera japonica* thunb and *Castanopsis hystrix*. Antivir. Res..

[100] MacRae W.D., Hudson J.B., Towers G.H.N. (1989). The antiviral action of lignans. Planta Med..

[101] Charlton J.L. (1998). Antiviral activity of lignans. J. Nat. Prod..

[102] Kajunguri D., Hargrove J.W., Ouifki R., Mugisha J., Coleman P.G., Welburn S.C. (2014). Modelling the use of insecticide-treated cattle to control tsetse and *Trypanosoma brucei* rhodesiense in a multi-host population. Bull. Math. Biol..

[103] Baker N., de Koning H.P., Mäser P., Horn D. (2013). Drug resistance in african trypanosomiasis: The melarsoprol and pentamidine story. Trends Parasitol..

[104] Kennedy P.G. (2013). Clinical features, diagnosis, and treatment of human african trypanosomiasis (sleeping sickness). Lancet Neurol..

[105] Urbina J.A. (2015). Recent clinical trials for the etiological treatment of chronic chagas disease: Advances, challenges and perspectives. J. Eukaryot. Microbiol..

[106] De Souza V.A., da Silva R., Pereira A.C., de A. Royo V., Saraiva J., Montanheiro M., de Souza G.H., da Silva Filho A.A., Grando M.D., Donate P.M. (2005). Trypanocidal activity of (−)-cubebin derivatives against free amastigote forms of *Trypanosoma cruzi*. Bioorg. Med. Chem. Lett..

[107] Schmidt T.J., Khalid S.A., Romanha A.J., Alves T.M.A., Biavatti M.W., Brun R., Da Costa F.B., de Castro S.L., Ferreira V.F., de Lacerda M.V.G. (2012). The potential of secondary metabolites from plants as drugs or leads against protozoan neglected diseases-part II. Curr. Med. Chem..

[108] Ohta K., Chen Y.L., Marumo S., Munakata K. (1969). Studies on piscicidal components of *Justicia hayatai* var *decumbens*. Part I. Isolation and piscicidal activities of justicidin A and B. Agric. Biol. Chem..

[109] Inamori Y., Kubo M., Tsujibo H., Ogawa M., Baba K., Kozawa M., Fujita E. (1986). The biological activities of podophyllotoxin compounds. Chem. Pharm. Bull..

[110] Lievens D., von Hundelshausen P. (2011). Platelets in atherosclerosis. Thromb. Haemost..

[111] Zhou L., Schmaier A.H. (2005). Platelet aggregation testing in platelet-rich plasma: Description of procedures with the aim to develop standards in the field. Am. J. Clin. Pathol..

[112] Siess W. (1989). Molecular mechanisms of platelet activation. Physiol. Rev..

[113] Chen C.C., Hsin W.C., Ko F.N., Huang Y.L., Ou J.C., Teng C.M. (1996). Antiplatelet arylnaphthalide lignans from *Justicia procumbens*. J. Nat. Prod..

[114] Wu C.M., Wu S.C., Chung W.J., Lin H.C., Chen K.T., Chen Y.C., Hsu M.F., Yang J.M., Wang J.P., Lin C.N. (2007). Antiplatelet effect and selective binding to cyclooxygenase (COX) by molecular docking analysis of flavonoids and lignans. Int. J. Mol. Sci..

[115] Hou R.C.W., Chen H.L., Tzen J.T., Jeng K.C.G. (2003). Effect of sesame antioxidants on LPS-induced NO production by BV2 microglial cells. Neuroreport.

[116] Lee D.Y., Seo K.H., Jeong R.H., Lee S.M., Kim G.S., Noh H.J., Kim S.Y., Kim G.W., Kim J.Y., Baek N.I. (2012). Anti-inflammatory lignans from the fruits of *Acanthopanax sessiliflorus*. Molecules.

[117] Rao Y.K., Geethangili M., Fang S.H., Tzeng Y.M. (2007). Antioxidant and cytotoxic activities of naturally occurring phenolic and related compounds: A comparative study. Food Chem. Toxicol..

[118] McGahon A., Bissonnette R., Schmitt M., Cotter K.M., Green D.R., Cotter T.G. (1994). BCR-ABL maintains resistance of chronic myelogenous leukemia cells to apoptotic cell death. Blood.

[119] Luo J., Hu Y., Kong W., Yang M. (2014). Evaluation and structure-activity relationship analysis of a new series of arylnaphthalene lignans as potential anti-tumor agents. PLoS ONE.

[120] Momekov G., Konstantinov S., Dineva I., Ionkova I. (2011). Effect of justicidin B—A potent cytotoxic and pro-apoptotic arylnaphtalene lignan on human breast cancer-derived cell lines. Neoplasma.

[121] Momekov G., Yossifov D., Guenova M., Michova A., Stoyanov N., Konstantinov S., Ionkov T., Sacheva P., Ionkova I. (2014). Apoptotic mechanisms of the biotechnologically produced arylnaphtalene lignan justicidin B in the acute myeloid leukemia-derived cell line HL-60. Pharmacol. Rep..

[122] Jin H., Yin H.L., Liu S.J., Chen L., Tian Y., Li B., Wang Q., Dong J.X. (2014). Cytotoxic activity of lignans from *Justicia procumbens*. Fitoterapia.

[123] Ilieva Y., Zhelezova I., Atanasova T., Zaharieva M.M., Sasheva P., Ionkova I., Konstantinov S. (2014). Cytotoxic effect of the biotechnologically-derived justicidin B on human lymphoma cells. Biotechnol. Lett..

[124] Siggers T., Chang A.B., Teixeira A., Wong D., Williams K.J., Ahmed B., Ragoussis J., Udalova I.A., Smale S.T., Bulyk M.L. (2012). Principles of dimer-specific gene regulation revealed by a comprehensive characterization of NF-κB family DNA binding. Nat. Immunol..

[125] Baba A., Kawamura N., Makino H., Ohta Y., Taketomi S., Sohda T. (1996). Studies on disease-modifying antirheumatic drugs: Synthesis of novel quinoline and quinazoline derivatives and their anti-inflamatory effect. J. Med. Chem..

[126] Ueng Y.F., Chen C.C., Chen C.F. (2000). Inhibition of benzo(a)pyrene hydroxylation by lignans isolated from *Justicia procumbens*. J. Food Drug Anal..

[127] Nebert D.W., Shi Z., Galvez-Peralta M., Uno S., Dragin N. (2013). Oral benzo[a]pyrene: Understanding pharmacokinetics, detoxication, and consequences-Cyp1 knockout mouse lines as a paradigm. Mol. Pharmacol..

[128] Shertzer H.G., Puga A., Chang C., Smith P., Nebert D.W., Setchell K.D., Dalton T.P. (1999). Inhibition of CYP1A1 enzyme activity in mouse hepatoma cell culture by soybean isoflavones. Chem.-Biol. Interact..

